# Supplementary material for: Neutrophil Extracellular Traps Promote the Development and Growth of Human Salivary Stones
Source: Cells. 2020 Sep 22;9(9):2139. doi: 10.3390/cells9092139 (PMC7564068; doi:10.3390/cells9092139)
Supplement: Supplementary file 1 [file cells-09-02139-s001.pdf]

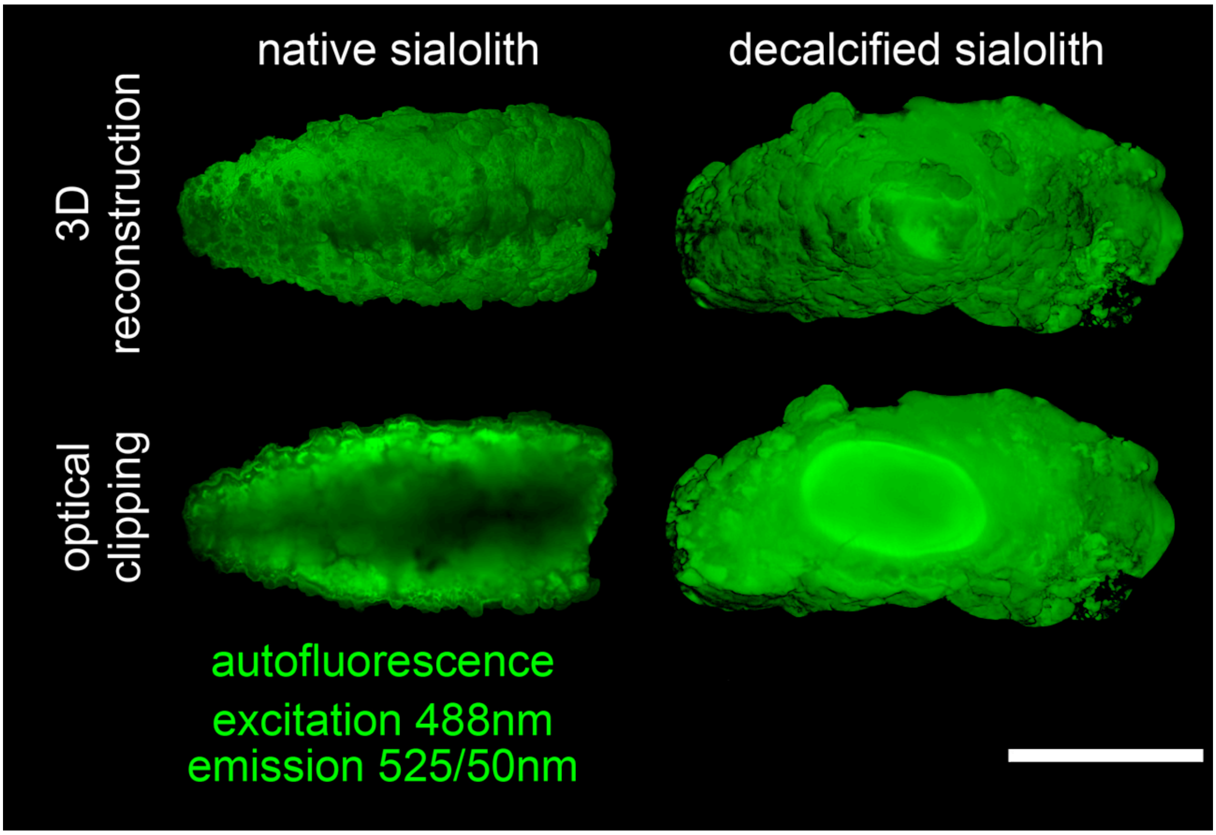

Figure 1 S (supplement). The center of the sialolith with a strong autofluorescence, discriminating the core from the outer layers.

Representative LSFM (Light sheet fluorescence microscopy) of a submandibular sialolith (n = 3). After decalcification, the strong central autofluorescence separated the core from the outer layers. Scale bar: 10 mm.
